# Influence of nature on spirituality and cognition: an examination of short-term exposure through video clips

**DOI:** 10.3389/fpsyg.2025.1498628

**Published:** 2025-05-19

**Authors:** Takechika Hayashi, Michio Nomura

**Affiliations:** ^1^Graduate School of Education, Kyoto University, Kyoto, Japan; ^2^Society for the Promotion of Science, Tokyo, Japan

**Keywords:** self-transcendence emotion, spirituality, nature, IAT, Japanese

## Abstract

**Introduction:**

Nature exerts a significant impact on spiritual experiences. While extended interactions with nature ranging from living in nature-rich neighborhoods to hiking through mountains for some time, have been found to promote spirituality and religiosity among individuals less is known about how its dual aspects (beautiful vs. threatening) and trait (dispositional) spirituality interact to shape these effects.

**Methods:**

In a controlled laboratory study, 57 Japanese university students (*M*age = 21.46 ± 2.60, 61.4% men) were exposed to natural scenes with contrasting emotional tones (positive and threatening) and completed self-report measures of spirituality and cognitive transformation alongside implicit association tests.

**Results:**

Results indicated that watching nature scenes promoted spiritual feelings and induced cognitive shifts in perceptions of self-size, time availability, and self-transcendence. Notably, participants viewing positive nature scenes reported higher spirituality scores than those exposed to threatening scenes. Crucially, trait spirituality emerged as a significant moderator; individuals with higher dispositional spirituality exhibited a more pronounced increase in state-level spirituality in both nature conditions compared to a neutral baseline.

**Discussion:**

These findings provide empirical support for theoretical perspectives suggesting that both positive and even adverse natural experiences, such as natural disasters, can enhance spiritual awareness by amplifying momentary shifts. The study underscores the importance of considering the dual aspects of nature when examining spiritual experiences and highlights the interplay between state and trait spirituality. These findings also underscore the need for more nuanced research on environmental influences on spiritual identity.

## 1 Introduction

The natural environment enhances a profound influence on human psychology, offering benefits that range from encouraging eco-friendly behaviors, such as making sustainable choices (Hinds and Sparks, 2008), to promoting subjective wellbeing (Mayer and Frantz, 2004). Beyond this, nature may also shape deeper psychological processes—such as one's sense of self, perception of time, and the awareness of interconnectedness—that can lay the groundwork for spiritual or transcendent experiences. For instance, perceiving oneself as part of a larger natural system has been linked to more empathetic attitudes (Zelenski et al., 2023), heightened attentional focus (Kaplan, 1995), and expanded time perception (Rudd et al., 2012). These shifts in cognition and emotion not only influence day-to-day wellbeing and behavior but can also foster feelings of awe or reverence, thereby leading us to consider the spiritual dimensions of nature's influence. Nature has long been recognized—both theoretically and empirically—as a potent source of spirituality and religiosity across diverse cultures. For instance, Kaplan and Kaplan (1989) and Watling (2013) argue that natural elements frequently inspire spiritual experiences, a notion that is especially evident in regions where nature worship (e.g., animism and manaism) positions nature as the primary object of faith. Complementing this perspective, research in the United States has shown that abundant natural amenities—such as beautiful landscapes and favorable weather—can serve as a spiritual resource by fostering a connection with the sacred, even effectively competing with organized religious institutions (Ferguson and Tamburello, 2015). Empirical studies further support these ideas: engaging in recreational activities in natural settings has been found to enhance spiritual wellbeing (Heintzman, 2000), and exposure to nature often evokes self-transcendent emotions, such as awe, which is widely recognized as a key facet of spiritual experience (Shiota et al., 2007). Together, these findings underscore the role of nature as not only an inspirational and spiritual alternative but also a fundamental component in the expression of religiosity across various cultural contexts.

In this study, we defined spirituality as the process of seeking and experiencing a connection to a transcendent dimension or power, which may involve emotional states such as awe and reverence and entail a deeper, cognitive-based sense of self-transcendence. Drawing on the Japanese Youth Spirituality Scale (Nigorikawa et al., 2016), we included reason to live (ikigai) and independence (a sense of autonomy or self-reliance) as facets of spirituality rather than mere indicators of wellbeing, given that they reflect an individual's perception of purpose and alignment with something greater than the self. Although spirituality and religion can overlap, this study distinguished religion as comprising organized doctrines, rituals, and institutional practices, whereas spirituality in our study refers to a personal, experiential process rooted in one's cultural context—such as the deep sense of connection to nature, ancestors, or a higher power—without requiring participation in formal religious structures.

Although spirituality could be understood as a quality or belief system that individuals foster overtime throughout their lives, experiences of nature exposure for shorter duration have been implied to be particularly effective in promoting spirituality. For example, hiking in mountainous areas has been shown to elicit feelings of self-transcendence, where individuals report a reduced focus on the self and an increased connection to a greater whole (Castelo et al., 2021). Similarly, experimental studies have demonstrated that viewing nature scenes for as brief as 2 min can induce a significant increase in feelings of awe, leading to perceived more available time, and a smaller sense of self, all of which are closely linked to spiritual experiences (Piff et al., 2015; Rudd et al., 2012). Thus, brief exposure to nature can be a quick and effective method for cultivating spiritual experiences. This aligns with theoretical perspectives that link awe and spiritual experiences as transformative states where perceptions of time and space are altered (James, 1902; Yaden et al., 2017).

**Figure 1:**
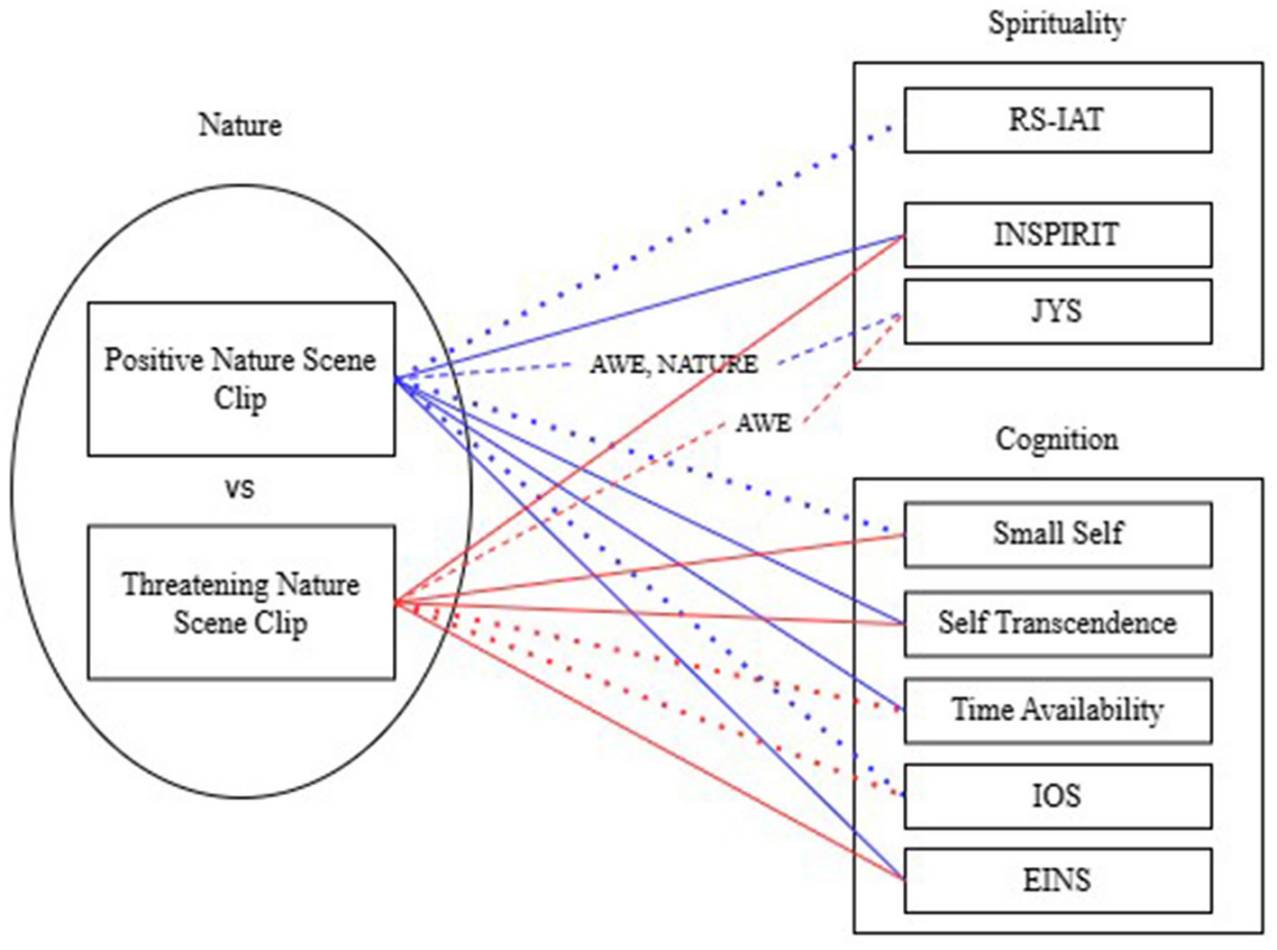
Framework and measurements of the study. RS-IAT, Religiousness/Spirituality Implicit Association Test; INSPIRIT, Index of Core Spiritual Experiences; JYS, Japanese Youth Spirituality Rating Scale; IOS, Inclusion of Others in the Self; EINS, Extended Version of Inclusion of Nature in the Self Scale. Lines show significant differences against the neutral condition. Solid line: significant; Dashed line: partially significant (at least one of the subscales are significant) with written subscales being significant; Dotted line: not significant; lines with text: differences between positive and threatening condition.

The current study used multiple measurements to comprehensively assess the diverse effects of nature on spirituality (Figure 1). Self-report scales and an implicit association test were used to assess participants' spirituality, and scales of cognitive transformation were used to assess their spiritual experiences. Moreover, although changes in state-level spirituality and cognitive changes related to spiritual experiences have been partially investigated in terms of the impact of short-term exposure to nature, how negative aspects of nature may influence these aspects of spirituality remain unclear. Thus, this study explored the altered sense of spirituality and cognitive transformations in the face of both beautiful and threatening nature conditions and state-level spirituality.

## 2 Methods

### 2.1 Participants

The participants were 57 Japanese-speaking students from Kyoto University (*M*age = 21.46, *SD* = 2.60, men = 35). Those who had citizenship in Japan and lived at least 18 years in Japan to adequately reflect spirituality within Japanese culture were included in the study. This study was approved by the Ethics Review Board of Kyoto University. All participants provided written informed consent before participating in the study. One of the questions in the first section of the questionnaire was used to check the attention of the participants to exclude those who randomly produced responses to the items. Only one participant missed the question, and the corresponding data were excluded using the listwise deletion method.

To ensure the statistical power of our study and accurately detect the effect size of interest, we conducted a priori sample size calculation using G^*^Power 3.1.9.7. Our main analysis was a multiple comparison of dependent variables (e.g., spirituality) among three different conditions. Using Holm's (1979) method, the alpha level that was used was one-third of 0.05 (α = 0.0167). Assuming a moderate effect size (*d* = 0.50), as per a previous study using the same stimuli (Gordon et al., 2017), and a power of 0.80, 57 participants were needed.

### 2.2 Procedures

The experiment employed a within-subjects design in which all participants experienced three conditions (neutral, positive, and threat). Each condition comprised a 2 min video, the Religiousness/Spirituality Implicit Association Test (RS-IAT; LaBouff et al., 2010), and the completion of a questionnaire. The participants watched a video on a 50-inch monitor in an experimental room with dimmed lights for an enhanced experience. Following each video, the participants performed the RS-IAT and then completed self-report scales assessing their emotional state, spirituality, and cognitive transformation in a randomized order to minimize potential biases associated with the sequence of the measures. To further mitigate procedural biases inherent in the within-subjects design, the order of the positive and threat conditions was counterbalanced across participants, while the neutral condition was consistently presented first to establish baseline scores with minimal bias. Additionally, short 2 min breaks were provided between each condition to reduce participant fatigue and minimize carryover effects from preceding conditions. Among the three conditions, the neutral condition served as a baseline for comparison and was strategically placed first to avoid the influence of nature video clips that are typically high in emotional arousal. After completing all conditions, the participants filled out measures of dispositional spirituality and provided demographic information, including age, gender, and religious affiliation.

### 2.3 Materials

#### 2.3.1 Stimuli

Only one video clip was used for each condition. The nature clips have been validated as triggers for self-transcendent emotions (Takano and Nomura, 2022) and used in previous awe studies. In the neutral video clip, a male narrator describes an automobile company. The positive nature clip was first used by Piff et al. (2015) and was a cut from the BBC's *Planet Earth* series comprising time-lapsed nature scenery, such as starry skies, an enormous iceberg, and misty forests. The clips in the threat condition comprised natural disasters including volcanic eruptions, avalanches, and tornadoes [modified from Gordon et al. (2017) by Takano and Nomura (2022)].

#### 2.3.2 Emotions

A method similar to that employed by Liu et al. (2023) was used as a manipulation check to assess whether each video evoked the appropriate emotional state. This involved calculating the average scores for awe, amusement, and wonders for the awe score; the average scores for fear, anxiety, and nervousness for the fear score; the average scores for amusement, joy, and warmth for the positive affect score; and the average scores for anger, sadness, and shame for the negative affect score.

#### 2.3.3 Spirituality

Two self-report scales were used to assess state spirituality. The Index of Core Spiritual Experiences (INSPIRIT; Kass et al., 1991) comprises seven items that assess the extent to which the respondent has experienced closeness with God as well as the belief that God dwells within. The measure is considered usable with Japanese participants. Example items include, “How strongly religious (or spiritually oriented) do you consider yourself to be?” (*strong, somewhat strong, not very strong, not at all*) and “How close do you feel to God?” (*extremely close, somewhat close, not very close, I don't believe in God*). The instructions clarify that God refers to any type of higher power/metaphysical entity beyond human understanding and the response should be based on the individual's own interpretation of God. Respondents are also asked about spiritual experiences they have had (e.g., “An experience of communication with someone who has died”).

The Japanese Youth Spirituality Rating Scale (JYS; Nigorikawa et al., 2016) was used to measure state spirituality rooted in the Japanese context. The scale comprises 27 items and five factors; however, to assess state spirituality, we used the subscales that assess harmony with nature (JYS-NATURE; e.g., “nature is an accepting entity”), connection with ancestors/roots (JYS-ROOTS; e.g., “feeling something has been passed down from ancestors”), and awe of the unseen (JYS-AWE; e.g., “I believe in something unseen”). Items are responded to using a seven-point scale ranging from 1 = “strongly disagree” to 7 = “strongly agree.”

To assess dispositional spirituality, the two other factors of the JYS assessing *ikigai* (meaning in life) (JYS-IKIGAI; e.g., “I am satisfied with the life I've lived so far”) and independence [JYS-INDEPENDENCE; e.g., “When trouble happens, I rely on people” (reverse score)] were used in addition to the three factors of the JYS mentioned above. These two factors were excluded from state-level spirituality measures since existential emptiness represented by meaning in life and independence are not usual practice of defining spirituality especially in Western point of view though, as we've mentioned in the introduction, it is suggested that the definition of spirituality expands to something that supports one's identity and resists existential anxieties. Items from the INSPIRIT that seemed to ask about trait-like characteristics of spirituality were also used (e.g., “How often have you felt as though you were very close to a powerful spiritual force that seemed to lift you outside yourself?”).

In addition to self-report measures of spirituality, we used the Religiousness/Spirituality Implicit Association Test (RS-IAT; LaBouff et al., 2010), an implicit association test specifically developed to effectively measure the participant's level of religiousness/spirituality. Since people may be motivated to present their personal spiritual beliefs while avoiding admissions that could cast their spiritual practices or beliefs which may or may not be rooted in a certain religion in a negative light, such as spiritual struggles or participation motivated by external factors (Abu-Raiya, 2017), it is reasonable to use implicit measures combined with explicit self-report scales. The *D*1 score was used to assess the strength of the links between participants and the concept of religiosity and/or spirituality. We used the experimental program “Open Source Implicit Association Test,” which was developed by Hussey and Pierce (2016) and uploaded on GitHub. The participants completed the RS-IAT on a laptop running PsychoPy (v2022.2.4). While religion comprises common rituals and practices, spirituality does not necessarily include them. Thus, in this study, we focused specifically on spirituality, which we defined as a sense of connection to something greater than oneself, often involving feelings of awe, transcendence, or interconnectedness.

#### 2.3.4 Cognitive transformation

For this study, we measured changes in cognition related to the perception of time and space, as well as the perception of the self and the external world/others using different scales. For measuring changes in time perception, the Time-Availability Index (TIME; Rudd et al., 2012) was used, which asks about the sensation of having more time available. The Perceived Self-Size Scale (SELF-SIZE; Bai et al., 2017) was used for measuring spatial cognition. The scale asks respondents to indicate their size by choosing from seven circles of different sizes. Furthermore, the Inclusion of Others in Self (IOS; Aron et al., 1992) and Extended Inclusion of Nature in Self (EINS; Martin and Czellar, 2016) were used as visual evaluation methods in which circles and bars are used to indicate how close the person feels to others and how close they feel to nature, respectively. Lastly, the Self-Transcendence Scale (ST; Castelo et al., 2021) was used to assess the relationship between the self and a greater existence (e.g., feeling that one's life is part of something larger).

### 2.4 Data analysis

To evaluate differences in the dependent variables (spirituality and cognitive transformation), multiple comparisons using *t*-tests were conducted among the three conditions (neutral vs. positive, neutral vs. threat, and positive vs. threat). Additionally, using dispositional-JYS and INSPIRIT scores, the interactions between these scores and the dependent variables were examined using a linear mixed model, and each of the five trait JYS factors was examined. All analyses were performed in R (ver. 4.2.2; R Core Team (2022) using the statistical package for multiple comparisons and lmerTest package (Kuznetsova et al., 2017) for linear mixed models.

## 3 Results

### 3.1 Emotions

Table 2 shows the results of the multiple comparisons of emotions across the three conditions. The participants felt more awe in both conditions compared to the neutral condition (neutral vs. positive: *t*_(55)_ = −12.81, *p* < 0.001, *d* = 1.71, 95% CI [−2.79, 2.04]; neutral vs. threat: *t*_(55)_ = −10.78, *p* < 0.001, *d* = 1.44, 95% CI [−1.84, −1.44]) and felt the most fear (neutral vs. threat: *t*_(55)_ = −10.877, *p* < 0.001, *d* = 1.45, 95% CI [−3.32, −2.29]; positive vs. threat: *t*_(55)_ = −8.45, *p* < 0.001, *d* = 1.13, 95% CI [−2.57, −1.58]), and negative emotions (neutral vs. threat: *t*_(55)_ = −5.41, *p* < 0.001, *d* = 0.72, 95% CI [−1.21, −0.56]; positive vs. threat: *t*_(55)_ = −4.34, *p* = 0.001, *d* = 0.58, 95% CI [−0.96, −0.35]) in the threat condition. Regarding positive emotions, there was no significant difference between the neutral and positive conditions (*p* = 0.22), but the scores were highest in the positive condition and significantly higher compared to the threat condition (*t*_(55)_ = 8.73, *p* < 0.001, *d* = 1.17, 95% CI [1.38, 2.20]).

### 3.2 Spirituality

Table 1 shows the results of the multiple comparisons of state spirituality across the three conditions. No differences were found between conditions in state spirituality as measured by the RS-IAT. However, the state INSPIRIT scores were the highest in the positive condition, whereas the mean score of the threat condition was higher than the neutral condition (neutral vs. positive: *t*_(55)_ = −8.02, *p* < 0.001, *d* = 1.07, 95% CI [−0.79, −0.47]; positive vs. threat: *t*_(55)_ = 3.31, *p* = 0.03, *d* = 0.44, 95% CI [0.08, 0.32]; neutral vs. threat: *t*_(55)_ = −5.31, *p* < 0.001, *d* = 0.71, 95% CI [−0.60, −0.27]). The JYS-NATURE scores were significantly higher in the positive condition than in the neutral and threat conditions (neutral vs. positive: *t*_(55)_ = −7.58, *p* < 0.001, *d* = 1.01, 95% CI [−2.30, −1.34]; positive vs. threat: *t*_(55)_ = 8.02, *p* < 0.001, *d* = 0.17, 95% CI [1.13, 1.89]; neutral vs. threat: *t*(55) = −1.49, *p* = 1.00, *d* = 0.20, 95% CI [−0.71, 0.11]). The JYS-AWE scores were significantly higher in the positive and threat conditions than in the neutral condition; however, there was no significant difference between the positive and threat conditions (neutral vs. positive: *t*_(55)_ = −7.56, *p* < 0.001, *d* = 1.01, 95% CI [−1.58, −0.92]; neutral vs. threat: *t*_(55)_ = −7.76, *p* < 0.001, *d* = 1.04, 95% CI [−1.74, −1.03]; positive vs. threat: *t*_(55)_ = −1.30, *p* = 1.00, *d* = 0.17, 95% CI [−0.35, 0.07]). Interestingly, no significant differences were observed between the three conditions for the JYS-ROOTS scores (*p* = 1.00).

**Table 1:** Descriptive statistics and multiple comparisons of state spirituality across the three conditions.

**Measure**	**Neutral (N)**		**Positive (P)**		**Threat (T)**		**Overall trend**
	***M***	***SD***	***M***	***SD***	***M***	***SD***	
RS-IAT	0.75	0.28	0.77	0.27	0.73	0.23	Not significant
INSPIRIT	1.53	0.36	2.16	0.72	1.95	0.73	N < P^***^, N < T^***^, T < P^*^
**JYS**							
Awe	2.80	1.49	4.05	1.36	4.18	1.24	N < P^***^, N < T^***^, P = T
Nature	3.16	1.94	4.98	1.39	3.51	1.57	N < P^***^, T < P^***^, N = P
Roots	2.48	1.57	2.61	1.59	2.53	1.66	Not significant

RS-IAT, Religiousness/Spirituality Implicit Association Test; INSPIRIT, Index of Core Spiritual Experiences; JYS, Japanese Youth Spirituality Rating Scale. ^*^p < 0.05; ^***^p < 0.001.

### 3.3 Cognitive transformation

Table 2 shows the results of the multiple comparisons of cognitive transformation across the three conditions. Self-Transcendence Scale (ST) scores and Extended Inclusion of Nature in Self (EINS) scores were significantly higher in the positive and threat conditions than in the neutral condition (ST: neutral vs. positive: *t*_(55)_ = −6.86, *p* < 0.001, *d* = 0.92, 95% CI [−1.39, −0.76], neutral vs. threat: *t*_(55)_ = −4.59, *p* < 0.001, *d* = 0.61, 95% CI [−1.05, −0.41]; EINS: neutral vs. positive: *t*_(55)_ = −11.44, *p* < 0.001, *d* = 1.53, 95% CI [−2.82, −1.98], neutral vs. threat: *t*_(55)_ = −11.05, *p* < 0.001, *d* = 1.48, 95% CI [−2.45, −1.70]), with no significant difference between the positive and threat conditions (ST *p* = 0.50; EINS: *p* = 0.42). The Time-Availability Index (TIME) scores were significantly higher in the positive condition than in the neutral and threat conditions (neutral vs. positive: *t*_(55)_ = −4.39, *p* = 0.001, *d* = 0.59, 95% CI [−1.06, −0.40]; positive vs. threat: *t*_(55)_ = 5.27, *p* < 0.001, *d* = 0.71, 95% CI [0.64, 1.42]), with no significant difference between the threat and neutral conditions (*p* = 0.53). The Perceived Self-Size Scale (SELF-SIZE) scores were significantly higher in the threat condition than in the neutral condition (*t*_(55)_ = 5.18, *p* < 0.001, *d* = 0.69, 95% CI [−0.40, 0.40]), whereas no significant difference was found between the positive and neutral conditions (*p* = 0.30). Additionally, there was no significant difference between the positive and threat conditions (*t*_(55)_ = 2.01, *p* = 0.64, *d* = 0.27, 95% CI [0.00, 0.86]). Lastly, the Inclusion of Others in Self showed no difference between the conditions (neutral vs. positive: *p* = 0.81; neutral vs. threat: *p* = 1.00; positive vs. threat: *p* = 0.46).

**Table 2:** Descriptive statistics and multiple comparisons of cognitive transformation across the three conditions.

**Measure**	**Neutral**		**Positive**		**Threat**		**Overall trend**
	***M***	***SD***	***M***	***SD***	***M***	***SD***	
**Emotion**							
Awe	2.69	1.20	5.11	1.24	4.96	1.21	N < P^***^, N < T^***^, P = T
Fear	1.86	1.10	2.58	1.55	4.67	1.49	N < T^***^, P < T^***^, N = P
Positive affect	3.29	1.43	3.85	1.48	2.12	1.38	T < P^***^, N = P, N = T
Negative affect	1.26	0.56	1.48	0.79	2.14	1.14	N < T^***^, P < T^**^, N = P
**Cognitive transformation**							
ST	2.37	0.18	3.44	0.20	3.06	0.19	N < P^***^, N < T^***^, P = T
IOS	2.04	0.15	2.46	0.22	2.05	0.18	Not significant
TIME	2.80	0.12	3.53	0.19	2.51	0.09	N < P^***^, T < P^***^, N = T
SELF-SIZE	3.30	0.20	2.71	0.24	2.29	0.22	N > T^***^, N = P, P = T
EINS	2.73	0.18	5.13	0.15	4.80	0.15	N < P^***^, N < T^***^, P = T

ST, Self-Transcendence Scale; IOS, Inclusion of Others in Self; TIME, Time-Availability Index; SELF-SIZE, Perceived Self-Size Scale; EINS, Extended Inclusion of Nature in Self. ^***^p < 0.001, ^**^0.001 ≤ p < 0.01.

### 3.4 Interaction between dispositional spirituality and conditions

We examined the effect of trait spirituality on state spirituality and cognitive changes using a mixed-effects model. For the trait spirituality score, we averaged the total score on the JYS (JYS-OVERALL) plus the JYS-NATURE, JYS-IKIGAI, JYS-AWE, JYS-ROOTS, and JYS-INDEPENDENCE subscale scores. We also used the average score of the items from the INSPIRIT that were thought to address trait content. Thus, a total of seven scores for trait spirituality were used, and a model was established for each. The experimental conditions (neutral, positive, and threat) and dispositional spirituality were set as fixed effects and participants as random effects.

**Figure 2:**
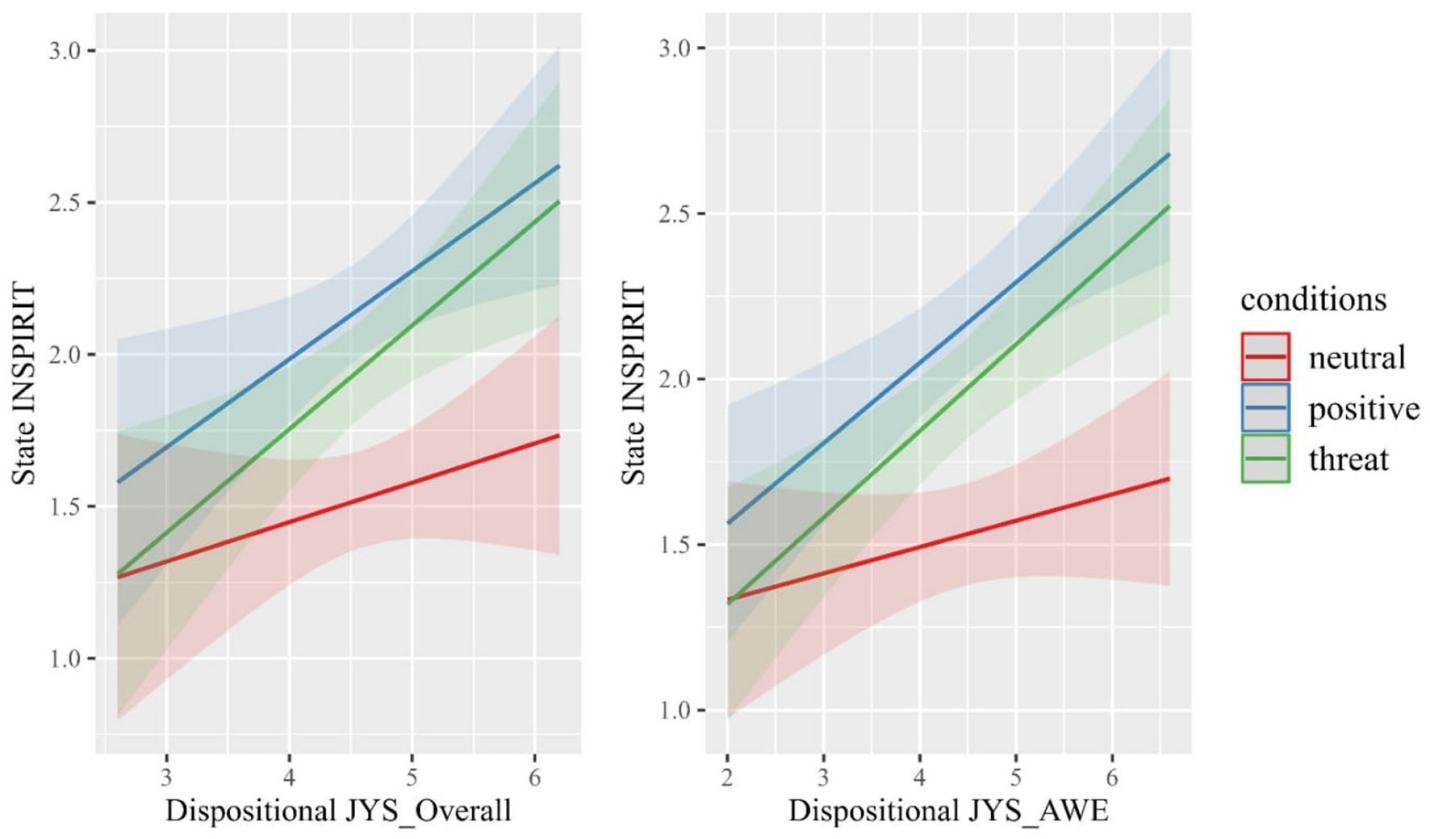
Interaction between scores of trait spirituality and experimental conditions on state spirituality scores. INSPIRIT, Index of Core Spiritual Experiences; JYS, Japanese Youth Spirituality Rating Scale.

The overall trend observed was higher scores on the dispositional scales were associated with higher scores on the state scales (For example, see Figure 2). Specifically, interactions were observed between JYS-OVERALL scores and state INSPIRIT scores in the threat condition (*b* = 0.21, *SE* =0.10, *t* = 2.07, *p* = 0.04) and state JYS-AWE in the positive condition (*b* = 0.33, *SE* = 0.21, *t* = 2.16, *p* = 0.03). Higher trait JYS-OVERALL scores in the threat condition increased the slope of improvement in state INSPIRIT scores, and higher trait JYS-AWE scores in the positive condition increased the slope of improvement. Moreover, interactions between trait INSPIRIT scores and state INSPIRIT scores were observed in both, positive (*b* = 0.48, *SE* = 0.16, *t* = 3.08, *p* = 0.003) and threat (*b* = 0.57, *SE* = 0.15, *t* = 3.72, *p* < 0.001) conditions. Compared to the neutral condition, higher trait INSPIRIT scores in the positive and threat conditions increased the slope of improvement in state INSPIRIT scores.

Subscales of trait spirituality, such as JYS_AWE, also predicted higher levels of improvement in state INSPIRIT scores. Interactions between JYS-AWE scores and state INSPIRIT scores were observed in both positive (*b* = 0.16, *SE* = 0.06, *t* = 2.64, *p* = 0.009) and threat (*b* = 0.18, *SE* = 0.06, *t* = 2.96, *p* = 0.004) conditions, and between state JYS-AWE scores and the positive condition (*b* = 0.35, *SE* = 0.13, *t* = 1.97, *p* = 0.05). Higher trait JYS-AWE scores in both positive and threat conditions increased the slope of improvement in state INSPIRIT scores, and only in the positive condition did it increase the slope of improvement in state JYS-AWE scores.

For the visual representations of full results on the interactions between dispositional spirituality and state spirituality/cognition, see the Supplementary material.

## 4 Discussion

In this study, we examined the effects of short-term exposure to both positive and threatening nature scenes on state spirituality and cognitive transformation, as well as the moderating influence of trait-level spirituality. Our findings reveal that natural scenes (positive and threatening conditions), compared to a neutral baseline, enhance state spirituality and induce cognitive changes; however, the outcomes differ depending on the emotional tone of the stimuli. Specifically, while the positive condition led to heightened experiences of awe and a greater sense of closeness with a transcendent entity, the threat condition elicited more pronounced fear and a reduction in perceived self-size. Importantly, individual differences in trait spirituality moderated these effects in a condition-specific manner, suggesting that inherent spiritual dispositions can amplify the impact of nature exposure on both spiritual and cognitive domains. This integrated pattern of results sets the stage for a more detailed discussion in the following sections.

### 4.1 Differences in state spirituality across conditions

Building on our overall findings, the data reveal that state spirituality increased significantly when participants were exposed to natural scenes compared to the neutral condition. Compared to the neutral condition, the participants reported a higher sense of closeness with God and awe of the unseen in the positive and threat conditions among a sample of Japanese university students. However, these condition effects did not emerge on the implicit spirituality measure, where scores remained statistically similar across all three conditions. One reason for why spirituality as measured by implicit association did not change might be that the set of words used did not sufficiently reflect spirituality in Japan. For example, the words used in the experiment were translations of the original English version, and terms like “agnostic” are not commonly heard in daily life in Japan, which could have influenced the results. Further studies should consider choosing words for the IAT that reflect the conceptual structure of spirituality specific to Japanese culture. Additionally, the participants reported that they felt harmony with nature to a greater extent in the positive condition than in the neutral and threat conditions, and experienced closeness with God to a greater extent in the positive condition than in the threat condition. These findings suggest that even short-term exposure to nature by simply watching video clips can make individuals feel more spiritual and develop a stronger sense of the power and presence of unseen entities.

Interestingly, state spirituality was shown to be a common feature in both, beautiful and threatening natural settings, although experiencing closeness with God was stronger in the positive condition than in the threat condition, indicating differences in intensity. The lack of a significant difference between the neutral and threat conditions with regard to harmony with nature suggests that these feelings were not evoked by the threatening natural videos. The videos viewed in the threat condition represented situations where one's life could be in danger (or at least suffer physical damage) if actually present and is represented by the high scores for feeling fear. This finding is consistent with that of the study by Takano and Nomura (2022), who used the same video clips in their study. Threatening stimuli activates the amygdala (e.g., Öhman, 2005), which in turn activates the sympathetic nervous system resulting in making our body ready to respond against the threatening stimuli. Gordon et al. (2017) revealed that higher levels of fear correlated with increased skin conductance and heart rate, which was the common outcome of heightened sympathetic nervous system activity. Therefore, it is possible that the participants' attention was directed more toward the fearful stimuli, given the physiological reaction, than the spiritual experience of closeness with God.

It indeed suggests that contact with nature can evoke a state of spirituality such that state spirituality assessed by INSPIRIT and JYS_AWE was higher in both beautiful and threatening natural condition compared to the neutral condition. However, interestingly, the INSPIRIT scores as well as the harmony with nature (JYS_NATURE) scores in the threatening condition were significantly lower than those in the positive condition, which calls for nuanced explanation about different intensity of two natural conditions. One possible explanation is because the threatening videos depicted scenarios where, if experienced firsthand, there could be a risk to life or physical harm, which induced strong feelings of fear which matches that the scores of fear emotion was higher in the threatening condition (*M*_*Neutral*−*Fear*_ = 1.86, *M*_*Positive*−*Fear*_ = 2.58, *M*_*Threat*−*Fear*_ = 4.67). Such fear likely resulted from the activation of the amygdala and subsequent sympathetic nervous system responses (Öhman, 2005; Gordon et al., 2017). Consequently, participants' attention may have been more focused on the fear response than on the spiritual experience.

### 4.2 Differences in cognition across conditions

In terms of cognitive transformation, our analyses indicate that natural scene exposure induces significant changes in self-perception and time awareness relative to a neutral baseline. Notably, the results showed that feelings of self-transcendence and closeness to nature were stronger in both natural conditions (i.e., positive and threat conditions) compared to the neutral condition. However, no significant differences were observed between the two natural conditions, demonstrating that viewing videos of nature scenery increased connectedness to nature and to something greater or larger than the self (e.g., all living things). In Castelo et al.'s (2021) study that examined the effect of exposure to nature on self-transcendence, the same trend was observed after completing hiking in nature settings. Our study replicates this effect with short-term virtual stimulation, which possibly emphasizes the significance of the conceptual aspect of nature rather than its material characteristics. Interestingly, no differences were observed across conditions in how close the participants felt to other human beings, which is consistent with van Cappellen and Saroglou (2012) who examined the effects of an awe-inducing nature clip on interconnectedness to friends and people in general. This implies that increased levels of spirituality elicited by nature enhances a sense of unity with larger entities, or “the world,” rather than fosters a sense of interconnectedness with other people. Because single-item measures have been criticized for their reliability, validity, and sensitivity (Allen et al., 2022), a more sophisticated and diverse measure that potentially includes multiple items of how close a person feels to others may more accurately capture the relationship between spirituality and connectedness to others.

Furthermore, participants reported the sensation of having more available time when watching the positive nature video than when watching the neutral or threat video, which is consistent with the findings of Rudd et al.'s (2012) study. They explained how an induced sense of awe predicted higher levels of time availability based on extended-now theory, which posits that the perception of time expands when the need for self-regulation occurs (Vohs and Schmeichel, 2003). It should be noted that this trend was not replicated in the threat condition in which participants felt fear and negative emotions. If self-regulatory emotional control changes time perception, the trend of increased feeling of perceived available time would have been observed in the threat condition as well, since participants would feel an urge to control induced emotions. One possible explanation is that we innately hold a vague feeling of impatience in life rather than a sense of having more available time. This impatience is reduced as one's focus moves from the self to others, which has been confirmed in studies on self-transcendence (Castelo et al., 2021; Stellar et al., 2017).

Regarding the perception of one's own size in space, participants perceived themselves smaller in the threat condition than in the neutral condition. Along with the simple reason that nature is far larger than a single human and is all-encompassing, experiences that heighten spirituality often involve a strong feeling of connection with larger entities (e.g., humanity as a whole, God), which is consistent with the results for self-transcendent feelings, possibly making one feel smaller (Yaden et al., 2016). Further investigation is thus needed to determine whether it is the sheer size of the viewed object or the self-transcendent emotions that influence the perception of one's size. However, a previous study (Piff et al., 2015) suggests that viewing nature involves more than the physical size of the object, incorporating interpretations of its power or presence, because the participants indicated that they felt smaller when viewing a large eucalyptus tree compared to large buildings.

### 4.3 Influence of dispositional spirituality on situational spiritual experiences

Finally, our mixed-effects analyses reveal that trait-level spirituality significantly moderates the state-level responses in both domains. Specifically, higher trait INSPIRIT scores were associated with greater increases in state spirituality in both the positive and threat conditions, while trait JYS-AWE scores showed a more pronounced moderating effect on the corresponding state measures in the positive condition. These findings underscore that individual differences in spiritual dispositions can amplify the effects of nature exposure, albeit in a condition-specific manner that is evident across both spiritual and cognitive transformations.

An individual's spirituality has both trait- and state-like aspects. On the one hand, it is regarded as the meaning system, a stable framework that develops throughout one's life and enables individuals to make sense of things, such as experiences that challenge one's existing point of view (Uwland-Sikkema et al., 2018). On the other hand, as examined in the present study and previous experimental studies (e.g., Crescentini et al., 2015, 2014; Miller et al., 2019), it is an emotional or cognitive response to what is happening in front of individuals, which changes by the minute. People who frequently feel spiritual emotions are expected to have more intense momentary reactions to spiritual experiences and matters, which are consistent with the results of the current study.

The results suggested that dispositional spirituality positively predicted state spirituality, including the state of awe of the unseen across three conditions. However, this interaction was confirmed only in the positive or threat conditions and depending on the combination of the measures of trait and state spirituality. For example, the interaction between the trait of connection with ancestors/roots and the state of self-transcendence was only observed in the threat condition. The more the participant felt connected with their ancestors and was aware of their roots at trait level (i.e., higher levels of spirituality), the higher the tendency for self-transcendence when watching videos of a threatening situation in nature.

People living in regions hit by natural disasters generally become spiritual/religious, with this effect persisting across generations (Bentzen, 2019). Japan is frequented by natural disasters, especially earthquakes and tsunami; it is thus reasonable that people in Japan would inherit various kinds of ideas through experiencing natural disasters and the following narratives about the reason it happens or lessons to learn told by people around individuals as much as appreciating beautiful aspect of nature. Thus, watching a video clip comprising natural disaster scenes might have contributed to participants identifying with their ancestors, thereby evoking a sense of connection with past and future generations and with humanity as a whole (i.e., increasing the state of self-transcendence).

### 4.4 Limitations and future directions

Our study has several limitations. The participants were all university students, which limits the generalizability of the results. Considering that one's spirituality may be associated with age or developmental stage, it would be important to replicate the study with different age groups. For example, older adults are more likely to think about their mortality and experience the loss of loved ones, which can lead to more chances of increasing spirituality. Moreover, spirituality scales developed in the context of Japanese culture confirm the importance of harmony and integration with nature as dimensions of spirituality, underscoring them as essential components of Japanese spirituality (Nigorikawa et al., 2016; Takeda et al., 2007). Considering that Japan frequently experiences natural disasters, and that individuals living in regions hit by natural disasters become more spiritual or religious (Bentzen, 2019), this study could have simply examined the uniqueness and commonalities of spirituality in a sample of Japanese university students. Diverse measures of spirituality, including implicit measures and performance, should be considered in future studies. It is also hoped that advancing analyses for each factor and item within a single scale will lead to the accumulation of knowledge about the processes that could be considered the core of spirituality.

## 5 Conclusion

Overall, this study revealed that short-term exposure to nature enhanced spirituality in a sample of Japanese university students. Different types of nature evoked different levels of spirituality and cognitive changes, and high trait spirituality predicted a greater increase in state spirituality. By refining the definition of spirituality from a psychological perspective on religion and spirituality, future research is expected to provide new insights.

## Data Availability

The raw data supporting the conclusions of this article will be made available by the authors, without undue reservation.
