# Supplementary material for: Influence of nature on spirituality and cognition: an examination of short-term exposure through video clips
Source: Front Psychol. 2025 May 19;16:1498628. doi: 10.3389/fpsyg.2025.1498628 (PMC12127365; doi:10.3389/fpsyg.2025.1498628)

Supplementary Material

Influence of Nature on Spirituality and Cognition: An Examination of Short-Term Exposure Through Video Clips

***Takechika Hayashi, Michio Nomura**

*** Correspondence:** Corresponding Author: hayashi.takechika.88d@st.kyoto-u.ac.jp

1 Supplementary Figures of interaction between dispositional and state level of spirituality/religiousness.

Interaction between dispositional JYS_Overall (left) / JYS_AWE (right) and state INSPIRIT.

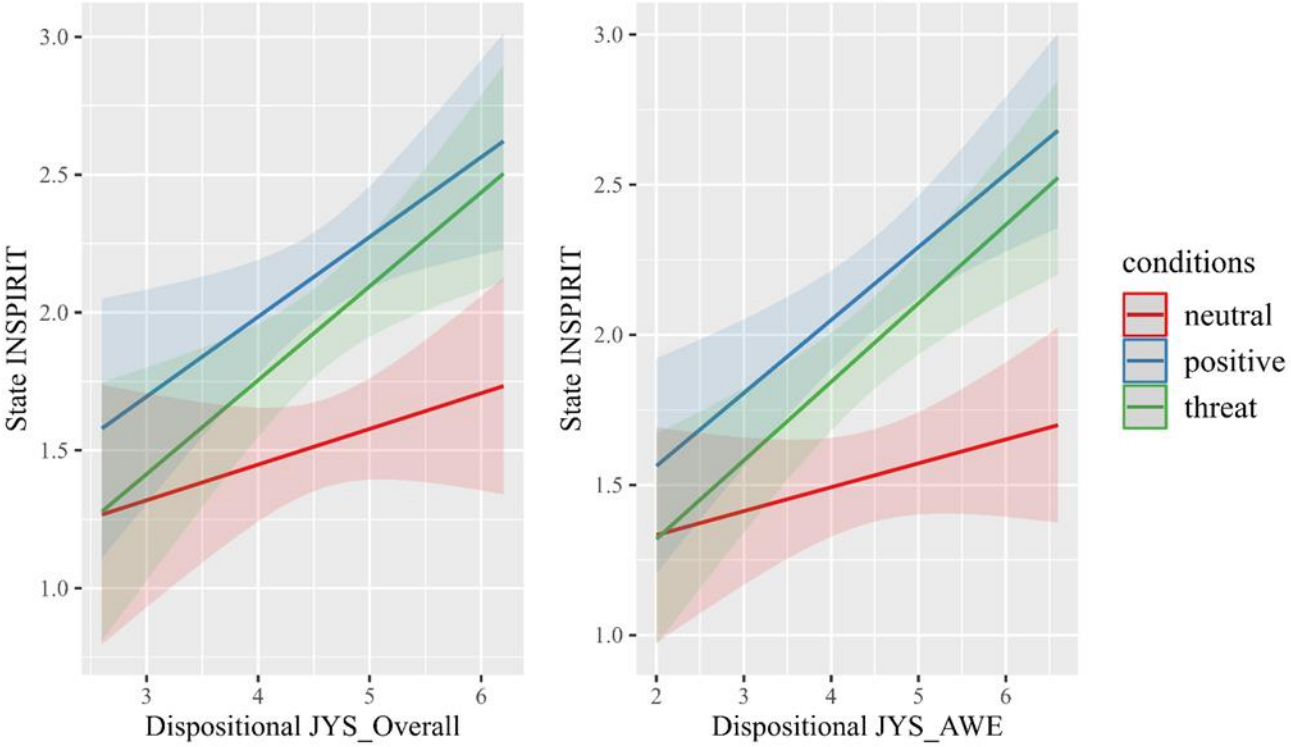

Interaction between dispositional JYS (the average score of all items across five subfactors) and state JYS-AWE (left) and state INSPIRIT (right).

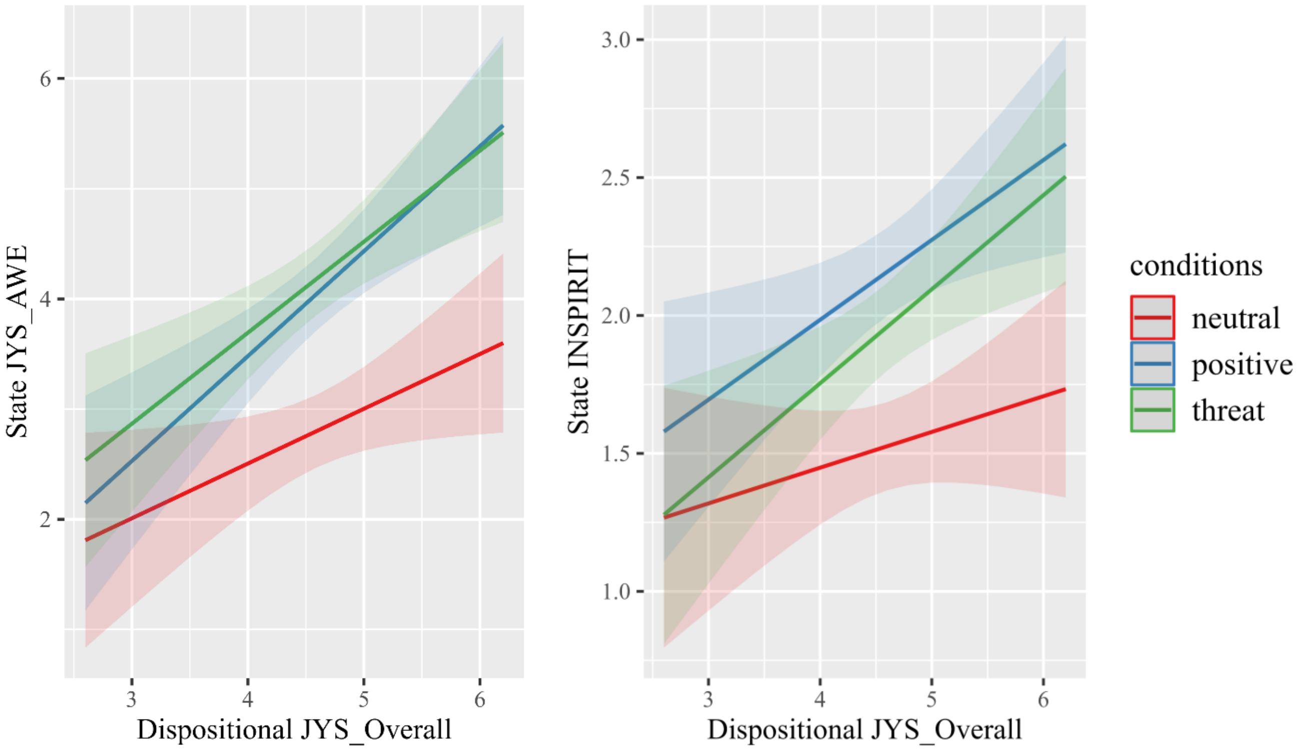
State JYS_AWE
6
4
2
3
4
5
6
Dispositional JYS_Overall
State INSPIRIT
3.0
2.5
2.0
1.5
1.0
3
4
5
6
Dispositional JYS_Overall
conditions
neutral
positive
threat

Interaction between dispositional and state INSPIRIT.

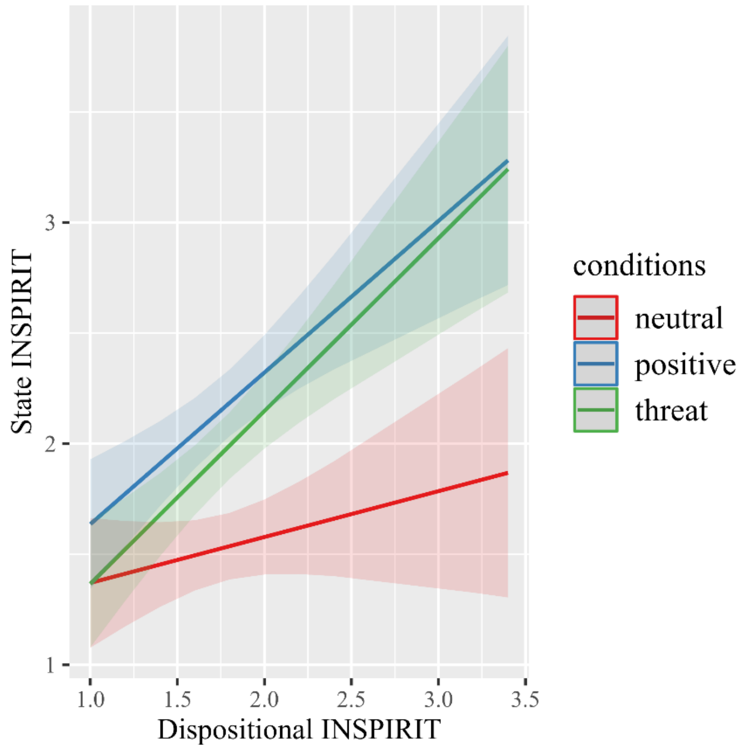

Interaction between dispositional JYS_NATURE and Negative Affect in Manipulation Check (left) and EINS (right).

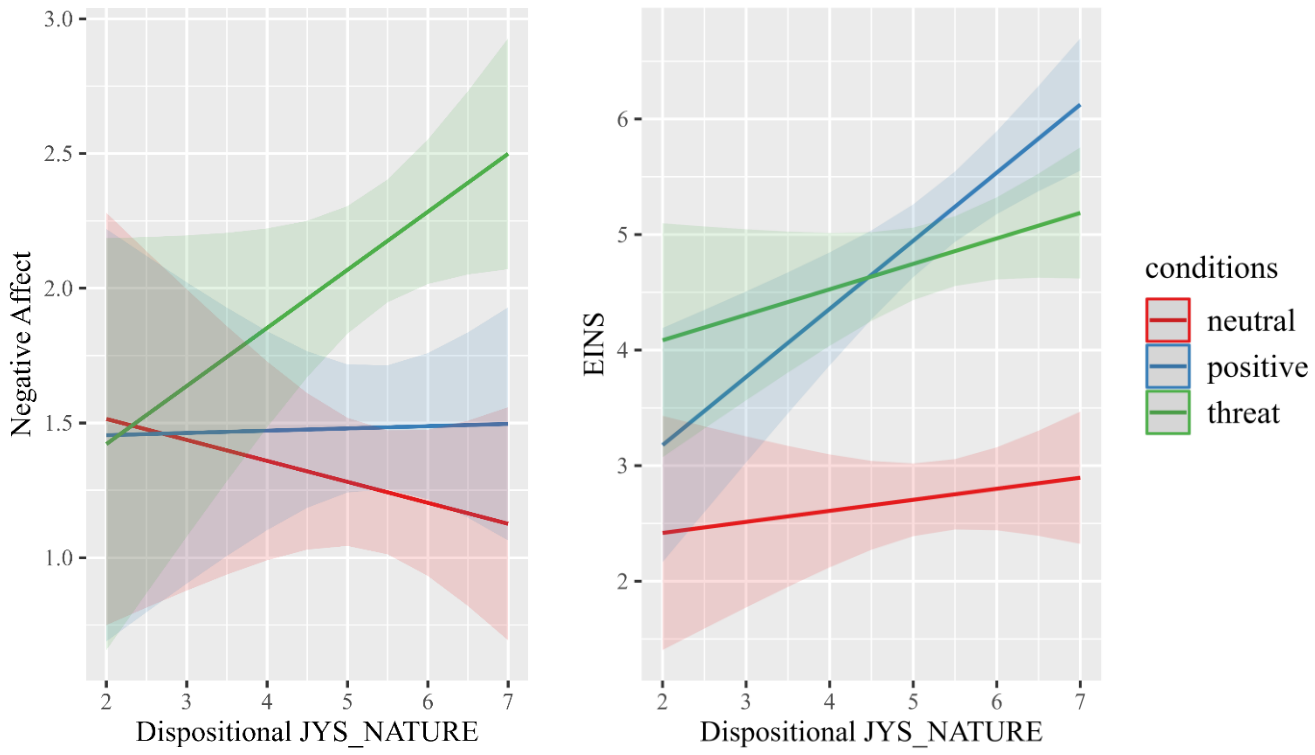

Interaction between dispositional JYS_AWE and State INSPIRIT (left) and State JYS_AWE (right).

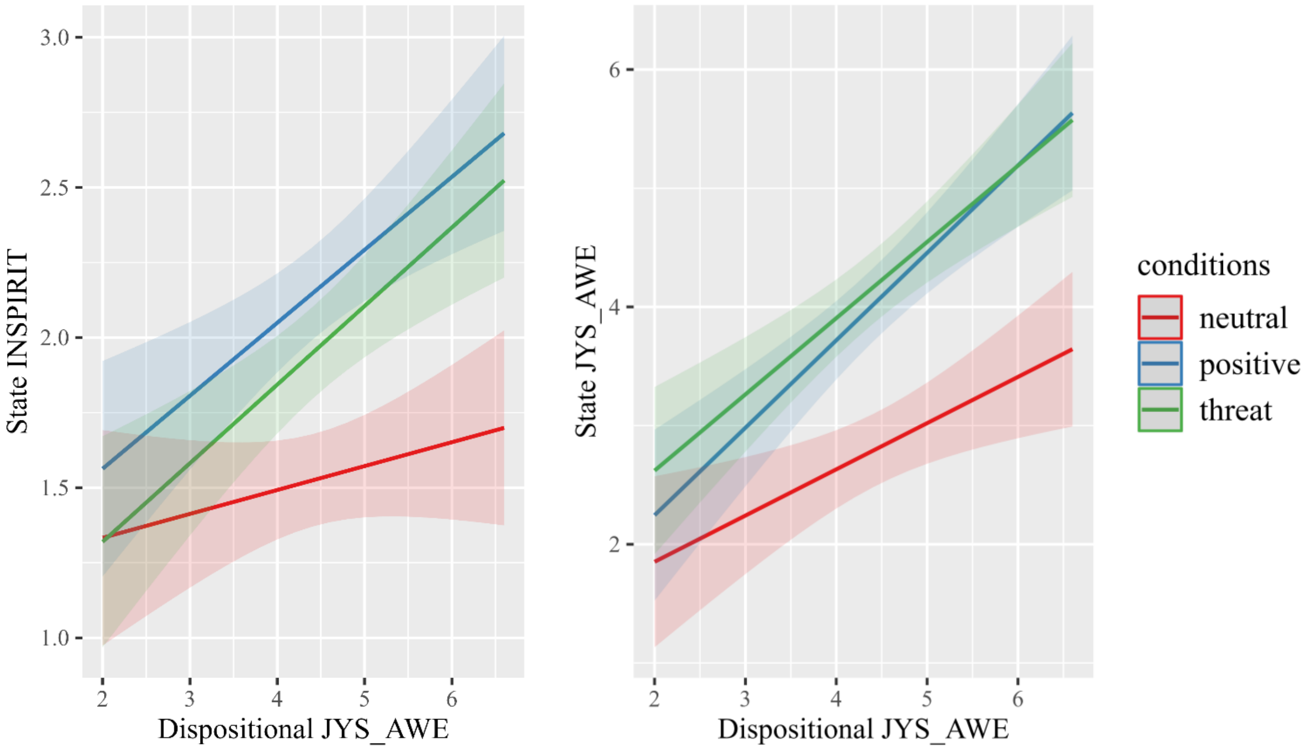

Interaction between dispositional JYS_ROOTS and ST. Interaction between dispositional

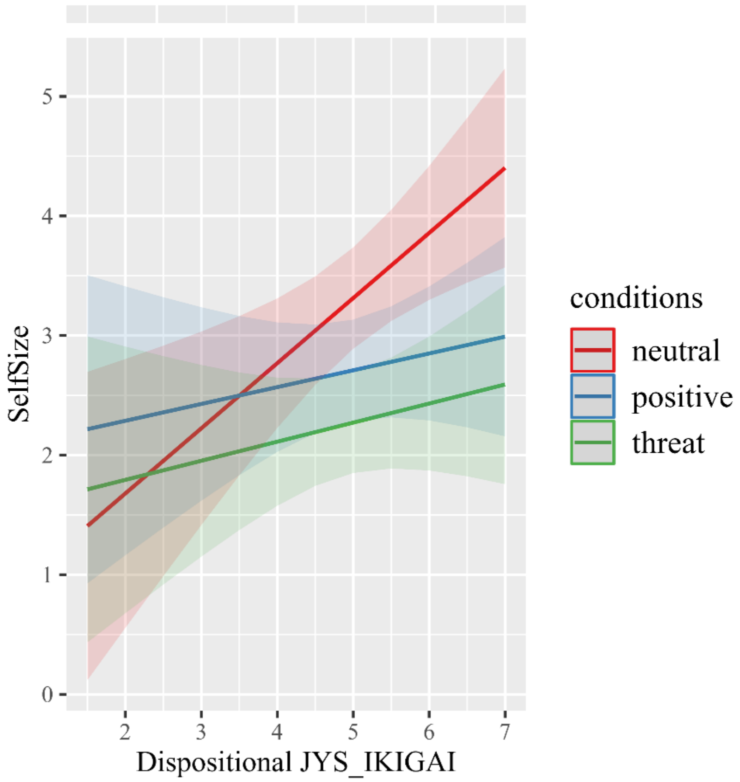

JYS_IKIGAI and Self-Size.

Interaction between dispositional JYS_INDEPENDENCE and RS-IAT (upper left), Awe (upper middle), Fear (upper right), Negative Affect (lower left), EINS (lower middle), and State JYS_NATURE (lower right).

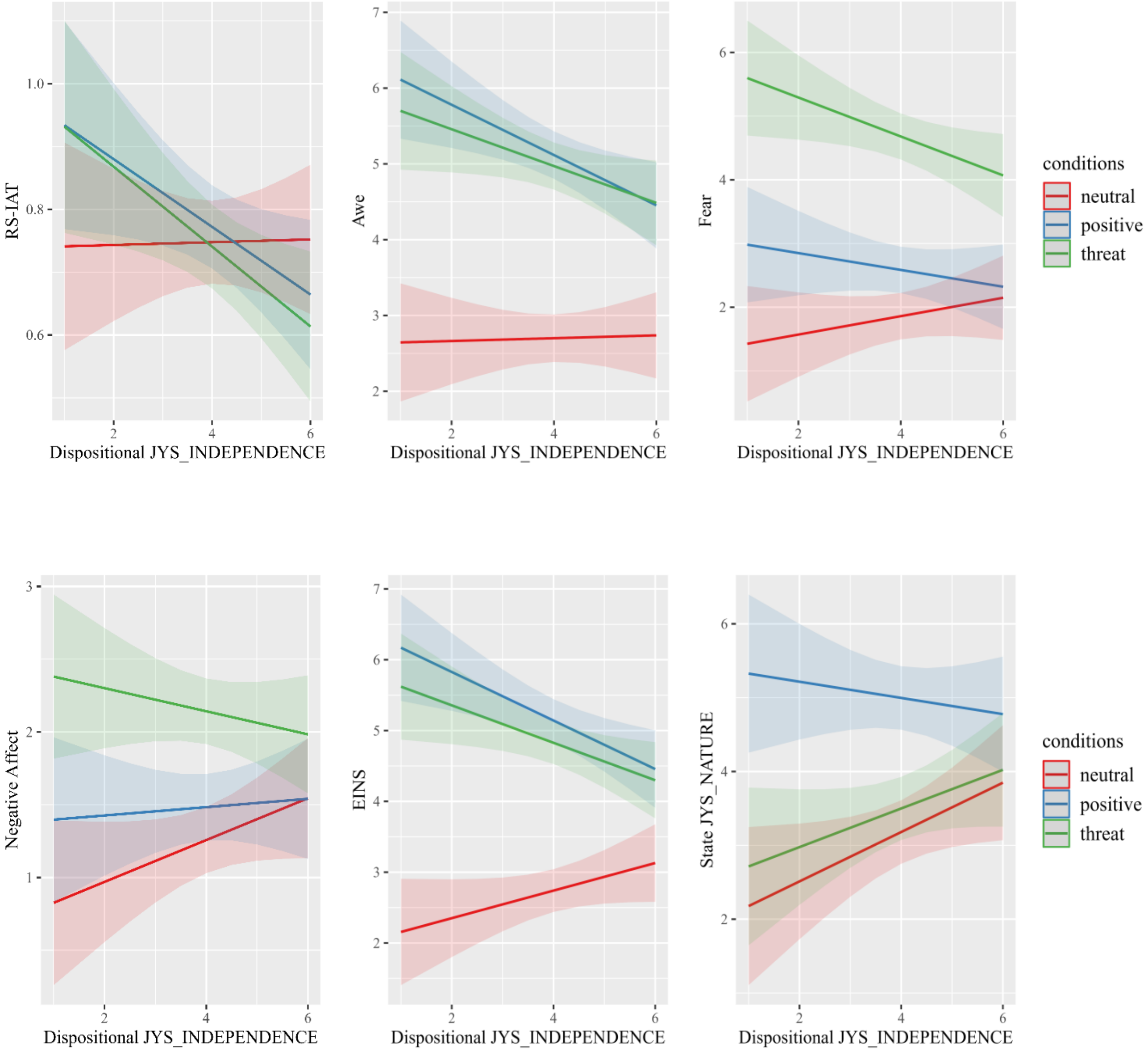

Supplement: Supplementary file 1 [file Data_Sheet_1.pdf]
